# The Territorial Force Nursing Service

**Published:** 1920-12-18

**Authors:** 


### THE MATRONS' AND SISTERS' DEPARTMENT.

### THE TERRITORIAL FORCE NURSING SERVICE.

THE veil of secrecy which hid the operations of the nursing sisters during the war has not yet been lifted. Lightning glimpses have come through from time to time, chiefly in the telling little narratives recounting deeds which have earned distinctions. But nothing approaching to a detailed history of "Nursing in the Great War" has yet been attempted. Hence we hail with particular pleasure the all too brief report furnished by Dame Maud McCarthy, G.B.E., of her own nursing Service handed in last week at a meeting of the Territorial Force Nursing Service Committee, City and County of London.

Dame Maud writes with restraint, but her pride in the Service of which she is Matron-in-Chief is not entirely banished by official reserve. It was a noble band of women, ably officered, admirably chosen, rising to heights of skill and endurance unguessed at by any at the outset of war. We can remember a time when much criticism was levelled against the preliminary organisation of the Territorial Force Nursing Service. The principle of selection in advance from the major training-schools of the kingdom, on the matron's recommendation, was strongly objected to in certain quarters. How splendidly it was vindicated by the event has become manifest.

The main body of Territorial nurses ready for service in 1914 amounted to a total of 2,738, of which number 2,116 nurses were required for 23 general hospitals and 667 to replace casualties. This was the backbone of the Service. As years went on, the principal matrons charged with this duty enrolled 5,357 more members. The total figures are 8,140, but the actual number who served was 7,117, for always they had a large body ready to join up as required.

Out of the 24 regularly organised hospitals of the Territorial Force, 10 were sent to France, 4 to Salonika, 1 to Malta, 1 to Egypt, 1 to Mesopotamia, and 1 to East Africa. All these as well as the Home Territorial Hospital were served by the Territorial nurses, and in addition the Service sent large reinforcements to the regular Army Nursing Service, and these were posted to casualty clearing-stations, ambulance trains, and barges and hospital ships.

The total of deaths was 48, of whom 5 were killed by enemy action; the rest, including 9 who died abroad, succumbed to illness. This low death-rate for a period extending over some five years reflects the highest credit on the organising ability of the heads of the Service. It is no higher in reality than what might be expected in a normal period out of an equal number of women engaged in ordinary occupations. When the prodigious toils of the war period be considered, the difficulties of transport, the improvised nurses' quarters, the many privations and dangers of war, nothing surprises more than to learn how few nursing sisters died in the course of their duties.

Yet perhaps it is after all more surprising still to learn that only 7 out of 7,000 were dismissed as "unsuitable." The art of selection has indeed been brought to a high pitch, and the art of training also, when but one in a thousand enrolled when increasing scarcity of nurses at home restricted choice, should have proved a failure.

Dame Maud herself is filled with admiration at the fine qualities which manifested themselves in those under her command. They preserved under all circumstances and difficulties a very high standard of nursing. This was expected of them, and the honour of the schools was safe in their hands. But they proved equal to many quite unaccustomed tasks. They were employed in surgical teams, had charge of wards where new forms of treatment were being carried out, took over small units and field ambulances in the very forward areas, managed a general hospital for the Portuguese, where they gave a fine object-lesson to some astonished gentlemen in the things British women could carry through; and, in fact, distinguished themselves under the most varied and bewildering experiences.

It is not merely their ability which stirs the admiration, it is their qualities of heart, their unstinted devotion to their patients and to the sorrowing relatives which move the emotions. When we thank God for victory, and not a day should pass without thanksgiving, let us thank Him for the quality of British nurses.

